# Reducing HPV Viral Burden in Men: A Synergistic Approach Using Pidotimod and Prophylactic Vaccination

**DOI:** 10.3390/microorganisms14061318

**Published:** 2026-06-12

**Authors:** Claudio Ucciferri, Livia Moffa, Giuseppe Vittorio De Socio, Katia Falasca

**Affiliations:** 1 Infectious Disease Clinic, Department of Medicine and Surgery, University of Perugia, 61123 Perugia, Italy; 2 Clinic of Infectious Diseases, Department of Medicine and Science of Aging, “G. d’Annunzio” University Chieti-Pescara, 66100 Chieti, Italy

**Keywords:** HPV clearance, adjuvant therapy, immunostimulant

## Abstract

Human papillomavirus (HPV) infection remains a major global health challenge, particularly when persistent high-risk genotypes lead to oncogenic progression. While prophylactic vaccines are effective, their role in accelerating the clearance of existing infections is still being explored. This study aimed to investigate the potential efficacy of adjunctive Pidotimod therapy combined with the nonavalent HPV vaccine in reducing persistent genotypes and promoting clearance in men. This retrospective pilot study included 23 HIV-negative men with anal and/or genital HPV infections. Participants were divided into two groups: 7 received the standard nonavalent HPV vaccine alone (control), and 16 received oral Pidotimod (800 mg twice daily for 10 days surrounding each vaccine dose) in addition to the vaccine (treatment). HPV genotyping (28 types) was performed at baseline and 12 months using real-time PCR. At 12 months, the HPV-negative conversion rate was 62.5% in the Pidotimod + vaccine group compared to 28.6% in the control group (*p* = 0.19). While this primary difference in total clearance was not statistically significant due to the limited sample size, the treatment group showed a substantial per-patient reduction in the number of persistent genotypes, decreasing from a mean of 2.75 ± 2.05 to 0.50 ± 0.82, compared to a decrease from 3.43 ± 2.37 to 1.86 ± 1.07 in the control group. The Pidotimod group achieved a significantly lower number of persistent genotypes at 12 months compared to the control group (*p* = 0.008, Mann–Whitney U test). Additionally, the use of pre-exposure prophylaxis (PrEP) was significantly associated with a lower rate of HPV clearance (12.5% vs. 73.3%, *p* < 0.01). Adjunctive therapy with Pidotimod suggests a promising trend in facilitating the reduction in HPV strain burden when combined with the HPV vaccine in men. While larger prospective studies are needed to confirm these effects, this exploratory approach could represent a promising immunomodulatory strategy for managing multiple and persistent HPV infections, even in high-risk groups such as PrEP users.

## 1. Introduction

Human papillomavirus (HPV) is one of the most prevalent sexually transmitted infections worldwide [1], with its unique oncogenic properties. Genital HPV types are typically categorized according to their epidemiological association as low-risk (LR-HPV) or high-risk (HR-HPV) genotypes. Although HPV infection has a high transmission rate, up to 90% of new HPV infections, including those with high-risk types, clear spontaneously or become undetectable within 24 months after infection [2].

In recent years, the use of immunomodulatory agents to enhance host defense mechanisms against HPV has gained significant attention. Among these agents, Pidotimod (PDT), a synthetic dipeptide (3-L-pyroglutamyl-L-thiazolidine-4-carboxylic acid), has emerged as a promising therapeutic adjuvant due to its well-characterized immunomodulatory properties [3]. Pidotimod has demonstrated its ability to enhance both cellular and humoral immune responses, with immunomodulatory activity affecting both innate and adaptive immunity [4]. Recent clinical evidence supports the therapeutic potential of PDT in HPV-related conditions. For instance, the combination of PDT with recombinant human interferon α-2b suppositories has been shown to effectively improve HPV negative conversion, accelerate vaginal microecology recovery, and modulate serum inflammatory responses in high-risk HPV patients following loop electrosurgical excision procedures [5], as well as to reduce the recurrence of female genital warts [6,7]. Furthermore, prospective studies have evaluated complementary treatment approaches in HPV-associated lesions, suggesting broader applications for enhancing viral clearance.

In clinical practice, PDT has shown efficacy in the prevention and treatment of various infectious conditions, including respiratory tract infections, demonstrating its potential utility in enhancing antiviral immunity [8,9,10]. The clinical usefulness of PDT and its role as an immunomodulator have been investigated for several decades, with a particular focus on its underlying mechanisms and its potential to enhance vaccine efficacy [3,11].

Given the persistent challenge of HPV clearance and the need for innovative therapeutic approaches, the role of immunomodulators in HPV-specific immunotherapy is increasingly recognized, especially in individuals who fail to mount a sufficient immune response. The integration of immunostimulant therapy with conventional preventative treatments may offer a synergistic approach to improving clinical outcomes in HPV-infected patients. This pilot study aims to investigate the exploratory effects of adjunctive PDT therapy in male subjects with anal or genital HPV infections who underwent nonavalent HPV vaccination.

## 2. Materials and Methods

### 2.1. Study Design and Population

This retrospective pilot study included 23 HIV-negative male patients who attended the outpatient sexually transmitted infection ambulatory clinic at SS Annunziata Hospital, University of Chieti, Italy, between July and December 2023. All patients underwent genital and anal swab collection for HPV infection.

Swabs in solution were analyzed for HPV-DNA by polymerase chain reaction, followed by type-specific hybridization. HPV detection and typing were performed using real-time PCR TOCE technology (Allplex HPV 28 detection, Seegene Inc., Seoul, Republic of Korea). Analysis was repeated at 12 months after complete dose vaccination.

Inclusion criteria were male adults aged between 18 and 65 who had sexual intercourse with men, with a confirmed HPV infection in the anal and/or genital area, and who had not received a previous HPV vaccination. Patients were excluded if they had incomplete medical records, refused HPV vaccination, or were HIV positive.

Data from 87 patients who were followed up for HPV infection during the study period were initially analyzed. Among these, 41 had not undergone any HPV treatment in the previous 6 months. Of the 31 patients who agreed to participate in the study, 23 completed the follow-up and were finally enrolled. Within this cohort, 16 patients accepted PDT supplementation in addition to the nonavalent HPV vaccine (treatment group), while the remaining 7 patients received the vaccine alone (control group). Patients were divided into two groups:

- Control Group: Received the standard nonavalent HPV vaccination at months 0, 1, and 6.
- Treatment Group: Received pidotimod 800 mg twice a day, on an empty stomach, starting three days before each scheduled vaccination dose for a total of 10 days, in addition to the nonavalent HPV vaccination at months 0, 1, and 6.

### 2.2. Data Collection

Data were extracted from electronic medical records, including patient demographics, comorbidities, previous history of warts, and treatment details.

The outcomes assessed were greater HPV negativity at 12 months, a greater reduction in the total number of strains, and fewer new genital/anal warts.

The secondary outcome was the presence of any factors affecting healing.

### 2.3. Sample and Data Collection

All specimens were collected by a trained medical practitioner using flocked swabs. Anal and genital sulcus samples were collected by rotating swabs 3 cm into the anal canal and around the coronal sulcus, respectively. Urethral mucosa specimens were obtained by performing three rotations within the first 2–3 cm of the urethral lumen.

Following collection, swabs stored in the preservation solution were processed for HPV-DNA testing. Genotyping was performed using the Allplex™ HPV28 Detection assay (Seegene Inc., Seoul, Republic of Korea), a multiplex real-time PCR platform based on TOCE™ technology. This method allows for the simultaneous detection and screening of 28 HPV genotypes (19 HR-HPV and 9 LR-HPV) in a single reaction, providing high sensitivity and specificity. This test was used to determine the presence of both low-risk genotypes (6, 11, 40, 42, 43, 44, 54, 61, 70) and high-risk genotypes (16, 18, 26, 31, 33, 35, 39, 45, 51, 52, 53, 56, 58, 59, 66, 68, 69, 73, 82).

Follow-up analysis was performed 12 months after the completion of the vaccination course.

### 2.4. Statistical Analysis

Data are expressed as mean ± standard deviation (SD) for continuous variables and were compared using Student’s *t*-test or analysis of variance, as appropriate, based on pre- and post-intervention data or based on different types of variables. Similarly, categorical variables were expressed as number and percentage and were analyzed using χ^2^ and Fisher’s exact tests, as appropriate.

A Mann–Whitney test was used to investigate the main effects and interaction effects of the intervention.

## 3. Results

A total of 23 HIV-negative males with anal/genital HPV positivity were enrolled: 16 in the PDT + HPV-vaccination group and 7 in the vaccination-only group. The median age was 40.1 +/− 9.5 years (41.5 vs. 37 *p* = ns). Eight patients were using PrEP, four in each group. At baseline, 16/23 (69.5%) patients were positive at the anal site and 14/23 (60.8%) at the genital site; 8/23 (34.7%) were positive at both sites (Table 1).

The most frequent genotypes at baseline were HPV-16 (in 9 males) and HPV-6 (in 6 males). HPV-6 strains were the most frequently detected strains at 12 months; the second most detected was HPV-58 (in 3 males). No new condylomas were detected after 12 months.

Clearance rates at 12 months were obtained in 12/23 males (52%) but were higher in patients treated with PDT + vaccination compared to vaccination-only patients (62% vs. 28%, *p* = 0.18) (Table 2).

**Figure 1:**
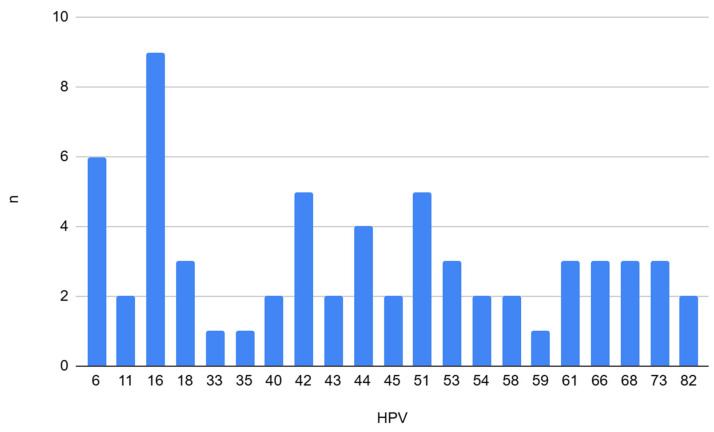
HPV detection at baseline.

**Figure 2:**
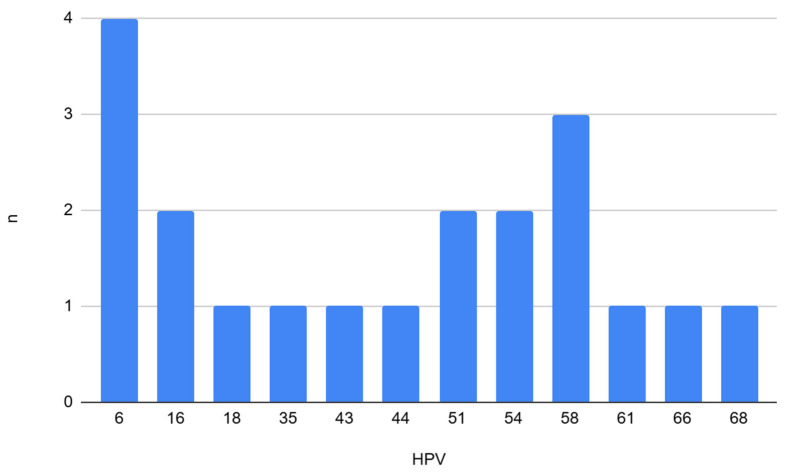
HPV detection at 12 months.

**Figure 3:**
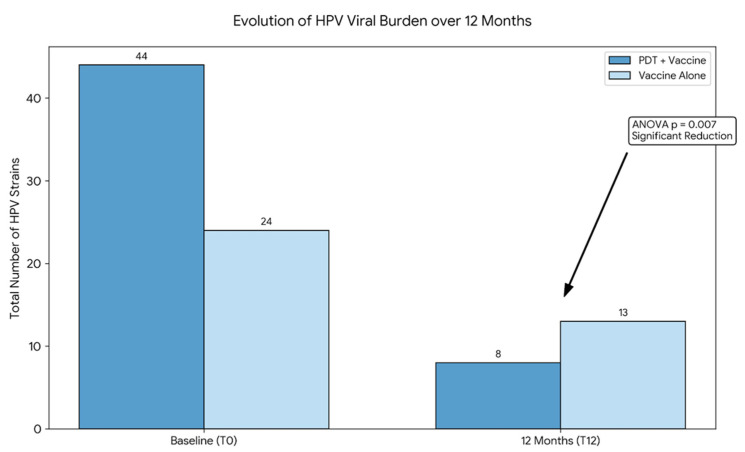
HPV evolution in Pidotomod + Vaccination group vs. Vaccination alone group.

Regarding the burden of HPV strains, a significant reduction in the number of HPV types was observed in the PDT group from baseline to 12 months (44 strains detected vs. 8 strains detected). The vaccination-only group showed 24 strains detected at baseline vs. 13 at the end of the observation period (Figure 1 and Figure 2). A significant correlation between PDT use and the number of strains detected at 12 months was demonstrated (*p* < 0.009) (Figure 3). Also, a significant reduction in the number of HR-HPV genotypes was observed in the PDT group vs. the control group from baseline to 12 months (*p* < 0.01), but no significant difference was shown in LR-HPV.

Baseline comparability analysis showed no statistically significant differences between the Pidotimod and control groups regarding age (*p* = 0.284) or the number of baseline HPV genotypes (mean 2.75, median 2.0 vs. mean 3.43, median 3.0, respectively; Mann–Whitney U = 47.0, *p* = 0.556). However, at 12 months post-treatment, patients in the Pidotimod group exhibited a significantly lower number of persistent HPV genotypes compared to the control group (mean 0.50, median 0. vs. mean 1.86, median 2.0, respectively; Mann–Whitney U = 18.5, *p* = 0.008). The use of PrEP negatively correlated with complete clearance at 12 months (12.5% vs. 73.3% *p* < 0.01). All HPV-58 strains detected at 12 months were found in PrEP users. PDT treatment was an independent and significant predictor of HPV strain reduction (*p* < 0.01), while confirming that PrEP use was a significant risk factor for viral persistence (*p* < 0.001). No adverse events were reported in the study.

## 4. Discussion

Our preliminary results suggest that adjunctive treatment with PDT combined with the nonavalent HPV vaccine may facilitate a reduction in the number of persistent HPV genotypes in HIV-negative men with anal and genital infections. Although the complete clearance rate in the PDT plus vaccine group was higher than that observed in the vaccine-only group (62.5% vs. 28.6%), the difference did not reach statistical significance (*p* = 0.19) due to the limited sample size. Notably, a statistically significant reduction in the number of HPV genotypes per patient at 12 months was observed in the PDT group compared with controls. While a decrease in individual genotypes was documented, PDT also emerged as an independent predictor of this reduction. Nonetheless, these data suggest that PDT could potentiate immune-driven suppression of persistent HPV types, which aligns with previous findings demonstrating enhanced immune activation and diminished HPV persistence following immunostimulatory interventions [5,12].

This finding aligns with emerging evidence supporting immunostimulants’ ability to enhance both innate and adaptive immunity, including dendritic cell maturation and T-helper 1 differentiation, which are critical for effective viral clearance. Such effects have been documented in respiratory tract infections [3,8,13] as well as HPV-related conditions, where immunomodulation reduces genital wart recurrence and improves post-surgical clearance [5,6,7]. Furthermore, prior studies also indicate that pidotimod can enhance vaccine efficacy and mitigate adverse effects when administered alongside vaccination [14,15]. The observed reduction in the individual count of oncogenic HPV genotypes in our treatment group preliminarily supports the role of PDT in facilitating the clearance of high-risk viral strains, which are strongly implicated in carcinogenesis. This potential effect may also share functional similarities with the immunomodulatory mechanisms of imiquimod, a topical agent widely used to treat condylomata acuminata [16,17].

Interestingly, HPV types 6 and 58 persisted more frequently despite treatment, which is consistent with data suggesting that certain HPV subtypes may exhibit differential clearance dynamics [1] and higher diffusion in the MSM population [18,19,20]. A key finding of our study is the strong negative correlation between PrEP use and complete HPV clearance [12.5% vs. 73.3%]. Notably, all HPV-58 strains detected at 12 months were found exclusively in PrEP users. This persistence is likely related to behavioral risk factors, such as increased exposure without barrier protection, rather than direct pharmacological effects of PrEP itself [18,20]. PDT remained a significant predictor of strain reduction even when accounting for PrEP use. The clinical implications of this finding warrant further investigation, especially considering the widespread use of PrEP and its potential influence on HPV epidemiology within this population [18]. The higher prevalence of PrEP users in the control group (57% vs. 24%) represents a potential confounding factor, as PrEP use is often associated with higher rates of sexual exposure and potential re-infection, and this could be the factor that contributed to the lower clearance rate observed in this group.

Current knowledge suggests that, although up to 90% of new HPV infections resolve naturally within two years, additional immunostimulatory therapies such as PDT may accelerate this process and improve long-term outcomes in populations with compromised immune responses or high viral loads (5). This hypothesis aligns with prior studies in women, where PDT combined with recombinant human interferon α-2b has been shown to effectively improve HPV-negative conversion and reduce the recurrence of female genital warts.

Overall, our study highlights the exploratory therapeutic potential of integrating immunomodulators with prophylactic vaccination to manage persistent HPV infections, especially in patients mounting suboptimal responses to vaccination alone. However, several critical limitations must be acknowledged. First, the retrospective, non-randomized design inherently introduces selection bias regarding treatment allocation, and the small sample size (N = 23) restricts the statistical power of our primary clinical endpoint. In order to try to limit some of these retrospective biases, the patients were all followed by the same center with strict inclusion criteria for age between 18 and 65 years and confirmed HIV-negative status. Finally, there is a lack of long-term follow-up to assess clinical outcomes such as lesion or condyloma recurrence. Given the small sample size, the non-randomized design, and the lack of statistical power for the primary endpoint of complete HPV clearance, these results must be interpreted with caution as strictly exploratory and hypothesis-generating. Consequently, future larger prospective randomized controlled trials are mandatory to validate these preliminary findings and further control for these confounders while elucidating the mechanisms underlying the synergistic effect of Pidotimod and HPV vaccination.

## 5. Conclusions

In conclusion, our preliminary findings suggest that Pidotimod is well-tolerated and may serve as a potential adjunctive immunomodulator alongside the nonavalent HPV vaccine, helping to reduce the number of persistent HPV genotypes per patient in men. Furthermore, our results underscore the critical role of behavioral and exposure-related factors; notably, PrEP use strongly correlated with viral persistence. Nonetheless, the observed reduction in individual genotype counts at 12 months suggests that target immunomodulation warrants further investigation. These findings contribute a preliminary step to the growing body of evidence exploring complementary approaches to manage persistent, multiple HPV infections in clinical practice, pending validation from larger, prospective randomized controlled trials.

## Figures and Tables

**Table 1:** Baseline characteristics.

Baseline Characteristic	Pidotimod + Vaccine (n = 16)	Vaccine Alone (n = 7)	*p*-Value
Mean Age (years)	41.6± 9.2	37.0 ± 10.1	ns
Mean Genotype Count (per patient)	2.75 ±2.05	3.43 ± 2.37	ns
Multiple Infection ($>1$ genotype)	11/16 (68.8%)	5/7 (71.4%)	ns
High-Risk HPV Presence	13/16 (81.2%)	7/7 (100.0%)	ns
Anal Infection Site	11/16 (68.8%)	5/7 (71.4%)	ns
Urethral Infection Site	7/16 (43.8%)	3/7 (42.9%)	ns
History of Condylomas/Warts	4/16 (25.0%)	2/7 (28.6%)	ns

**Table 2:** HPV outcome.

Group	Number of Patients	Mean Age (Years)	HPV Negative Rate at 12 Months (%)	Viral Burden Reduction at 12 Months (%)	Clearance of HR-HPV Strains at 12 Months (%)	PrEP Users (%)
Pidotimod + HPV Vaccine	16	41.5 ± 9.2	62.5%	81.8%	87%	24%
HPV Vaccine alone	7	37.0 ± 10.1	28.6%	45.8%	36%	57%

## Data Availability

The raw data supporting the conclusions of this article will be made available by the authors on request.

## Abbreviations

The following abbreviations are used in this manuscript:

HPV	Human papillomavirus
PDT	Pidotimod
PrEP	HIV pre-exposure prophylaxis
MSM	Men who have Sex with Men
